# Combining a leadership course and multi-source feedback has no effect on leadership skills of leaders in postgraduate medical education. An intervention study with a control group

**DOI:** 10.1186/1472-6920-9-72

**Published:** 2009-12-10

**Authors:** Bente Malling, Lene Mortensen, Thomas Bonderup, Albert Scherpbier, Charlotte Ringsted

**Affiliations:** 1 Department of Human Resources, Aarhus University Hospital, Skejby, Aarhus, Denmark; 2 Department of Internal Medicine, Regional Hospital Viborg, Denmark; 3 Department of Human Resources The North Denmark Region, Aalborg, Denmark; 4 Institute for Education, Faculty of Health, Medicine and Life Sciences, Maastricht University, Maastricht, The Netherlands; 5 Centre for Clinical Education, Copenhagen University and the Capital Region, Rigshospitalet, Copenhagen, Denmark; 6 Correspondence address: Mollerupvej 5, DK 8600 Silkeborg

## Abstract

**Background:**

Leadership courses and multi-source feedback are widely used developmental tools for leaders in health care. On this background we aimed to study the additional effect of a leadership course following a multi-source feedback procedure compared to multi-source feedback alone especially regarding development of leadership skills over time.

**Methods:**

Study participants were consultants responsible for postgraduate medical education at clinical departments. Study design: pre-post measures with an intervention and control group. The intervention was participation in a seven-day leadership course. Scores of multi-source feedback from the consultants responsible for education and respondents (heads of department, consultants and doctors in specialist training) were collected before and one year after the intervention and analysed using Mann-Whitney's U-test and Multivariate analysis of variances.

**Results:**

There were no differences in multi-source feedback scores at one year follow up compared to baseline measurements, either in the intervention or in the control group (p = 0.149).

**Conclusion:**

The study indicates that a leadership course following a MSF procedure compared to MSF alone does not improve leadership skills of consultants responsible for education in clinical departments. Developing leadership skills takes time and the time frame of one year might have been too short to show improvement in leadership skills of consultants responsible for education. Further studies are needed to investigate if other combination of initiatives to develop leadership might have more impact in the clinical setting.

## Background

Postgraduate medical education (PGME) usually takes place at many clinical departments in both university and non-university hospitals [1-3]. The increasing demands to PGME in the clinical departments at hospitals have made it necessary to appoint leaders of PGME at every training site [1,3].

The expectations of the leader of PGME in the clinical departments are high and they vary across stakeholders according to their position in the department [3]. Although the various stakeholders have only limited knowledge of the role of leaders in PGME, they suggest formal leadership education to meet the expectations [3].

Although it has recently been questioned it is still the general opinion that education of leaders results in improvement of leaders' performance [4-7], especially if the content of the leadership courses relates to the organisation the leader works in [5,6,8]. Many authors have suggested the use of a combination of methods to develop leadership skills [4-9]. McKimm used a combination of a leadership course and mentoring in a program for leaders in health and social care education [4]. Mintzberg advocated for a combination of personal development, leadership courses and experience by linking the leadership courses to practice in real or simulated situations [8]. In other organisations than health care it has been suggested to combine leadership courses with multi-source feedback (MSF) or other initiatives supposed to initiate personal development in coherence with development of the organisation [7,9].

We have previously demonstrated that MSF used by consultants responsible for PGME at clinical hospital departments resulted in clear and concrete plans for improvement of management skills, while plans for development of leadership skills and hence personal development were scarce and less concrete. These difficulties in formulating goals and plans for personal development were interpreted by stakeholders as a need for further initiatives to support leadership development [10].

On this background we aimed to study the effect of a leadership course following a MSF procedure compared to MSF alone especially regarding development of leadership skills over time.

## Methods

### Context of the study

Postgraduate medical education in Denmark is governed by the Danish National Board of Health. In clinical departments participating in PGME it is mandatory to appoint a consultant responsible for education (CRE). A number of different PGME programmes run in each clinical department and the CRE is thus responsible for a highly diverse group of young doctors in specialist training (trainees).

### Study design

The study was a non-randomised intervention study with a control group. The intervention was participation in a leadership course designed for CREs in clinical departments. Both participants in the intervention group (I-group) and the control group (C-group) went through a MSF procedure including personal feedback at baseline (MSF-I). Only the I-group participated in the leadership course following the baseline MSF procedure. One year after the intervention MSF procedure was repeated (MSF-II) in both the I-group and the C-group.

Effect was measured by change in MSF scores from CREs and from the following respondents: The head of department, the clinical consultants, and the trainees in the department. The CRE chose at least three consultants and three trainees to secure anonymity.

The study was presented to the ethical committee for Viborg and Aalborg County. In our jurisdiction studies of this kind do not need approval.

### Measuring instrument

The MSF instrument was developed for CREs in clinical departments and the validation has been described in a previous study [10]. The MSF instrument comprised 69 statements divided into four categories: 1) Technical skills, referring to the CRE's proficiency in specific methods, processes and techniques; 2) Human skills, including the ability to work with and through people to meet goals; 3) Citizenship behaviour, professionalism regarding interpersonal, organisational and job/task performance; 4) Administrative skills, involving knowledge of the organisation, planning, organising and coordinating the tasks of a CRE. Statements in technical skills, human skills and citizenship behavior comprise leadership while administrative skills refer to management. Each statement was scored on a seven-point Likert scale (One = "not at all" and seven = "always"). The option "not able to answer" was provided. An e-mail based electronic system (Enalyzer^®^) was used. The MSF instrument is provided in additional file 1.

A report on the CRE's self-ratings compared to respondents' rating was provided together with individual personal feedback by an experienced human resource consultant. The CRE was expected to make a plan for development of PGME in the department as well as for personal development of leadership skills based on the report and the feedback session.

### Participants

The investigations took place in the Northern Educational Region in Denmark, where a leadership course designed for CREs was offered to all CREs in the region. The Northern Educational Region comprise one third of the country. There are both university and non-university hospitals in the region and all medical specialties are represented. A convenience sample of CREs, who were included in two consecutive leadership courses constituted the I-group. CREs would have to come from clinical departments with more than three consultants in addition to the head of the department and more than three trainees to fulfill the inclusion criteria. The C-group consisted of 28 CREs from matched departments regarding size and specialty. None of the CREs in the C-group participated in the leadership course during the study period. All participants were contacted by phone and informed about the study and the MSF procedure. Confidentiality was guaranteed and participants were assured that it would be impossible to trace findings to individual participants, clinical departments or hospitals. After one year we excluded the CREs who had not completed either MSF-I or the leadership course as well as those who no longer held a position as a CRE.

### The course

The themes in the leadership course emerged from results of a needs assessment study [3], the general job description for CREs made by the Danish National Board of Health, and a literature study on leadership. The themes are shown in Table 1. The leadership course was divided into three modules: two three-day residential modules and a follow-up day. The course was held over a period of six months. Included in the course were two mandatory assignments: the first assignment was a general plan for one of the PGME programs in the department, the second was a five-year overall developmental plan for all PGME programs including possibilities in the department and possible barriers to implementation. The assignments were supposed to initiate reflection on own practice and hence combine the leadership course with real life experience.

**Table 1:** Themes in leadership course

Pedagogical knowledge	Principles of teaching and learning in clinical setting How to teach teachers and supervisors Various teaching methods Group dynamics
	
Organization of specialist training	Laws, rules and regulations Tasks and responsibilities of the CRE Handling problem-trainees
	
Educational culture	Factors influencing the educational culture How to influence the educational culture
	
Evaluation and quality assurance	In-training assessment Internal and external evaluation
	
Planning specialist training in the department	Effective teaching methods Integrating education and the working schedule Administrative tasks of a CRE
	
Supervision of supervisors	Consultation skills How to supervise peers Power relations in the department Appreciative inquiry and other methods
	
Implementation strategies	Change management Project management Implementation of new initiatives Strategic communication
	
Personal development	Personal development plan
	
Leadership in specialist training	Motivating and enabling Role modeling Conflict resolution
	
Research in medical education	Best evidence medical education Knowing the literature

Table 1 shows the themes in the leadership course designed for consultants responsible for education at departmental level (CRE).

The course was evaluated by the participants in four areas: 1) if the course met participants expectations, 2) if the content was relevant for the daily work as a CRE, 3) perceived benefits from attending the course and 4) perceived learning outcome. The four areas were scored on a four-point scale ranging from one = "not at all" to four = "to a high degree".

### Statistics

Mean of scores from CREs and MSF respondents were calculated. If a score was missing it was replaced by a mean of all other scores in the same category from the same respondent. A multivariate analysis was used to compare effect of a combination of MSF and a leadership course to the use of MSF alone. Covariant factors investigated were 1) respondent type (CRE or MSF respondents), 2) level of baseline MSF scores and 3) change in scores from baseline to one year after. Mann-Whitney's U-test was used to compare baseline scores in I-group and C-group and to compare completers and dropouts in the C-group. A p-value < 0.05 was considered significant.

## Results

**Figure 1:**
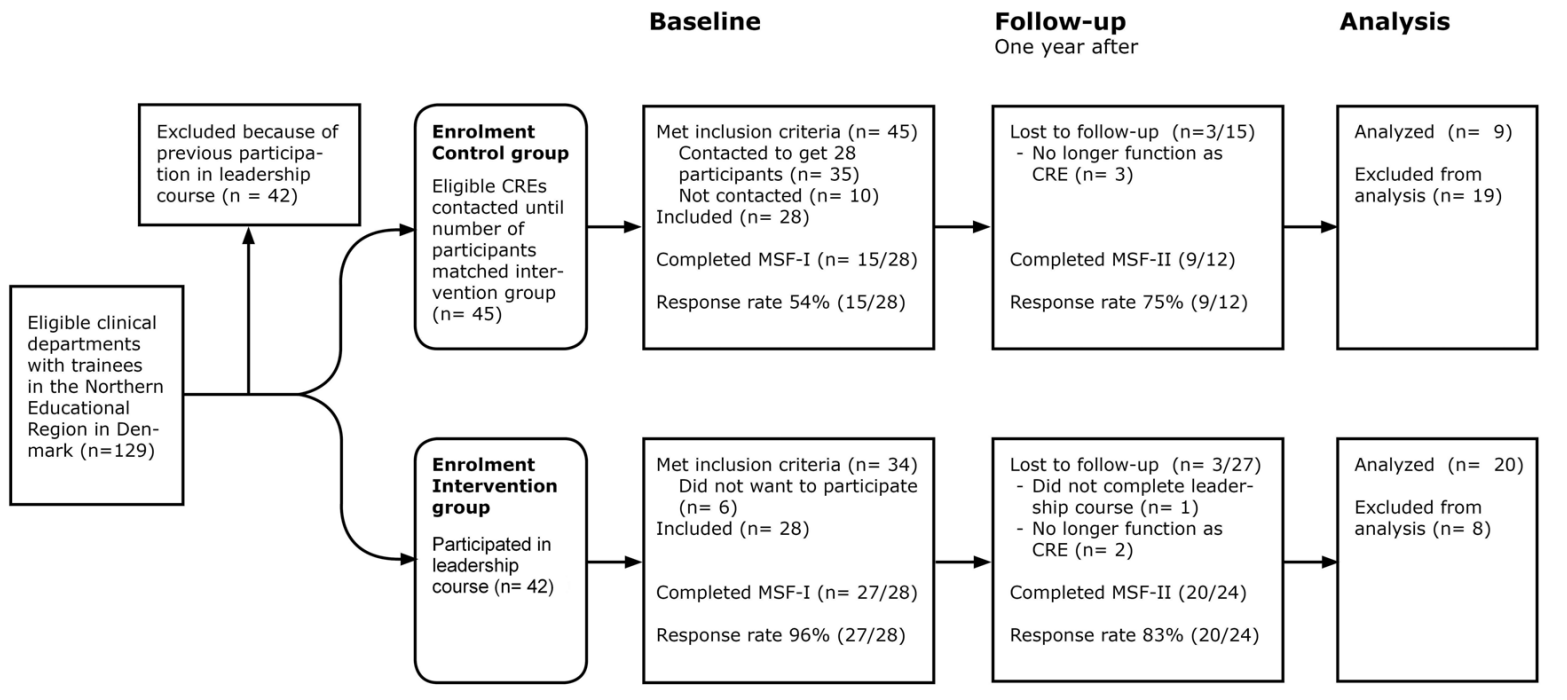
**Flowchart of the study**. Figure 1 shows the flowchart of the study. The number of consultants responsible for postgraduate medical education in clinical departments (CRE) included in the intervention and the control group. Intervention was a seven-day leadership course combined with a multisource feedback procedure before (MSF-I) and one year after (MSF-II) participation in the course. The control group went through both MSF-I and MSF-II. Response rate for CREs in both MSF-I and MSF-II is provided.

Figure 1 show the total number of CREs included in the study, the response rate and the number of CREs who dropped out or were excluded before MSF-II. Participants represented 16 of the 37 specialties in Denmark. Participants' evaluated the leadership course highly regarding relevance and perceived learning, see Table 2.

**Table 2:** Course evaluation

	Mean (SD)
The course met my expectations	3.4 (0.2)
Course content relevant for my daily work as a CRE	3.4 (0.2)
I benefited from attendance	3.3 (0.2)
I learned from participation	3.2 (0.2)

Evaluation of a leadership course rated by 40/42 consultants responsible for education at clinical departments. Evaluations are expressed as mean (SD) within four areas, and are rated by participants on a four-point scale ranging from 1 = "not at all" to 4 = "to a high degree".

Table 3 shows the MSF mean scores with SD of CREs and respondents from I-group and C-group respectively. Scores are shown for baseline and one year after the intervention. At baseline there were no differences in MSF scores between the I-group and the C-group in the scores from the CREs and respondents respectively. There was no difference in MSF scores for completers and dropouts in the C-group at baseline. The covariance analysis showed no statistical difference between I-group and C-group considering measurements at baseline and one year after (p = 0.149) when respondent type, level of baseline score and change in C-group from baseline to one year after was taken into account.

**Table 3:** Leadership performance

	Intervention group		Control group	
	CREs	Respondents	CREs	Respondents
Completed MSF-I (N)	27	288	15	131
Completed MSF-II (N)	20	217	9	72
				
**MSF score**	Mean (SD)	Mean (SD)	Mean (SD)	Mean (SD)
Baseline	5.5 (0.7)	5.6 (0.5)	5.4 (0.5)	5.5 (0.5)
One year after	5.8 (0.6)	5.6 (0.5)	5.5 (0.5)	5.7 (0.5)

Number of consultants responsible for education at clinical departments (CRE) and respondents in a multi-source feedback procedure (MSF) at baseline (MSF-I) and one year after the intervention group completed a leadership course (MSF-II). Mean MSF score (SD) for CREs and respondents are shown for the intervention and control groups (scale 1 - 7).

## Discussion

In this study the effect of a leadership course for consultants responsible for education in clinical departments following a MSF procedure was compared to MSF alone especially regarding development of leadership skills over time. Surprisingly, the study did not show the expected effect of a combination of a leadership course and MSF compared to MSF alone. In the following we first discuss various validity threats to our results including selection biases, problems of study design and instrumentation. Next we discuss various explanations of the un-expected results.

The use of a convenience sample of course participants may be a limitation in this study. The leadership course was offered to all CREs in the Northern Educational Region. Thus although all CREs in the region had the opportunity to participate in the course we cannot exclude a selection-bias in our sample, who represented those CREs who voluntarily signed up for the course. However, no difference in MSF scores was found between the I-group and C-group at baseline indicating that the groups were comparable.

The high drop-out rate especially in the C-group reflects the difficulties in recruiting busy clinicians to studies of this kind which included a time consuming MSF procedure. Another explanation to the high drop-out rate in the C-group might be that participants in this group were not supported in the same way as participants in the I-group who all passed through a course where they got help to solve various problems in their daily leadership of PGME in the departments. Therefore the motivation to complete the MSF-II procedure might have been much higher in the I-group than in the C-group who only got a phone call asking them to complete the MSF once more. Hence the C-group who completed the second MSF procedure might be the most enthusiastic CREs; they might be the CREs who actively seeks personal development. A selection-bias of this kind could have reduced the differences between the I-group and the C-group. However, at baseline, the MSF scores of participants from the C-group who completed both MSF procedures and scores of dropouts did not differ indicating that the influence of the high dropout might be small.

MSF in itself is an intervention intended to improve leaders' performance and hence the use of MSF as a measuring instrument might have been problematic. However, the results of the C-group do not indicate a major influence of the MSF procedure in itself. This is in accordance with our previous study where the development plans for CREs were not representative for areas needing improvement [10]. At baseline, both the scores of CREs and respondents are quite high and a ceiling effect cannot be ruled out.

Pooling the scores from respondents (the head of the department, the other consultants and the trainees in the department) into one score might have blurred the results, since it is well known that there are different perspectives on leaders' performance according to the position you hold in the organization (chief, peer, subordinate), and since the perception of the concept "good leader" varies among stakeholders [6,11]. According to a previous study, stakeholders' knowledge of the job as CRE is scarce and the expectations to the CRE as leader of the medical education in the department differs according to stakeholders' position in the department [3]. However, it has been shown that these different expectations did not result in differences in the various stakeholders' scoring in a MSF process [10]. We therefore feel confident in pooling the results from various stakeholders into one score of respondents for each CRE.

The second MSF was performed one year after the CREs participated in the leadership course. It is generally agreed that repeated MSF processes would eventually improve leaders' performance [11-14]. However, some describe that a time-span of one year is too short to detect any improvement in MSF scores [12,13]. It might therefore have been interesting to see if improvement in leadership skills of the CRE could be detected if the MSF procedure was repeated after 2-3 years, since developing as a leader is a time-consuming process and initiating changes in a highly bureaucratic organization is a slow process [6]. However, expanding the study period to more than one year might also have been problematic and might have resulted in even higher drop-out rates than was actually found.

The participants in our study came from both university and non-university hospitals and represented a variety of specialties. This supports the generalisability of the results within the domain of medical education. However, as both the instrument and the leadership course were tailored to CREs it is questionable whether results can be translated into health care leaders in general or to other domains of organisations.

Despite the limitations of the study we feel confident in concluding that the impact of leadership courses on performance in actual practice must be disputed. Previous studies have mainly described a positive effect of courses on leaders' knowledge and performance based on self-reported data [15,16]. The well-known gap between knowing and doing might be reflected in our study where the stakeholders' ratings do not indicate any improvement in leader performance, while at the same time participants in the study reported to have learned from the course. Some of the factors influencing this knowing-doing gap include physician barriers (peer influence and inertia), organizational barriers, and support/resource barriers [17,18]. In a previous study it was described how the CRE is considered to be in a weak position regarding influence and power, and how difficult it is for the CRE to take on a leadership role due to various environmental factors like peer influence, his place in the hierarchy and the general inertia existing for developmental initiatives in hospitals. [3]. When evaluating course effects factors such as support and follow-up from superiors, openness to change and new knowledge in the organization, stability and resources in the organization, and the possibility to practice what has been learned should be considered [15,18,19]. In addition when studying the effect of MSF procedures environmental factors like feedback orientation, organisational cynicism, how MSF fits into other developmental initiatives and stability in the organisation must be considered [11,13].

Further investigations are needed to explore the degree to which the clinical department supports the leaders of PGME and to investigate the relations between the culture in the department and the opportunity for CREs to display leadership skills.

## Conclusion

In this study the effect of a leadership course following a MSF procedure compared to MSF alone regarding development of leadership skills of consultants responsible for education at clinical department was investigated. Although participants reported to have learned from the leadership course no improvement was found. Various explanations like lack of organisational support and the culture in the departments might be speculated. Further studies are needed to investigate the role of environmental and other factors on leadership development in CREs.

## List of abbreviations

PGME: Postgraduate medical education; MSF: Multi-source feedback; CRE: Consultant responsible for education in clinical department; I-group: Intervention group; C-group: Control group; MSF-I: Multi-source feedback at baseline; MSF-II: Multisource feedback one year after intervention; SD: Standard deviation.

## Competing interests

The authors declare that they have no competing interests.

## Authors' contributions

BM, LM and TB made substantial contributions to the conception, design and the acquisition of data. BM analyzed the data. BM, LM, TB, AS and CR made substantial contribution to the interpretation of data and drafting of the manuscript. BM, LM, TB, AS & CR all made substantial contributions in critically revising the manuscript and content. All authors have given final approval of the version published.

## Authors' information

BM: MD, MHPE and associate professor in postgraduate medical education is program director for specialist training at Aarhus University Hospital, Skejby, Denmark.

LM: MD, PhD and associate professor in postgraduate medical education is consultant at the Department of Internal Medicine and program director at the Regional Hospital, Viborg, Denmark.

TB is consultant in human resources at Department for Human Resource, The North Denmark Region, Aalborg, Denmark.

AS: MD, PhD is professor of medical education and scientific director of the Institute for Education, Faculty Health, Medicine and Life Sciences, Maastricht University, Netherlands.

CR: MD, PhD, MHPE is professor of medical education and director of Centre for Clinical Education, Copenhagen University and Capital Region, Rigshospitalet, Denmark.

## Pre-publication history

The pre-publication history for this paper can be accessed here:

http://www.biomedcentral.com/1472-6920/9/72/prepub

## Supplementary Material

Additional file 1 The MSF measuring instrument. Click here for file
