# Supplementary material for: Combining a leadership course and multi-source feedback has no effect on leadership skills of leaders in postgraduate medical education. An intervention study with a control group
Source: BMC Med Educ. 2009 Dec 10;9:72. doi: 10.1186/1472-6920-9-72 (PMC2797774; doi:10.1186/1472-6920-9-72)
Supplement: Additional file 1 — The MSF measuring instrument. [file 1472-6920-9-72-S1.DOC]

### Appendix 1

The multisource feedback instrument developed for consultants responsible for postgraduate medical education in clinical departments.

### Leadership

### Technical skills

The CRE in your department

1. takes on the role as spokesman for trainees in educational matters
2. is a role-model in relation to education in the department
3. expresses his overall vision for education in the department
4. makes clear decisions
5. takes responsibility for education in the department
6. makes sure that the head of department supports the prioritization of education in the department
7. states that the quality of education in the department influences how attractive the trainees find the department
8. is goal-oriented
9. has managed to obtain influence on the organization of the work
10. announces if education is given too low priority
11. shows interest in each individual trainee
12. makes sure the consultants and other specialists in the department support educational matters
13. motivates the supervisors to maintain focus of the goals for education
14. is willing to guide and advice in educational matters

### Human skills

The CRE in your department

1. shows interest in the trainees as human beings not only as trainees
2. is sensitive to ideas and suggestions
3. sees resources in other people
4. calls for the opinion of others
5. has power to penetrate
6. shows that he likes the job as CRE
7. is ready to receive feedback himself

### Citizenship skills

The CRE in your department

1. signals commitment in his function as CRE
2. puts education on the department’s agenda with enthusiasm
3. constantly develops educational activities in the department
4. motivates doctors in the department to use all training possibilities
5. introduces all new doctors in the department
6. establishes network around education in the department
7. establishes contacts to create networks around education outside the department
8. works for a good educational environment
9. is visible to the doctors in the department in his function as CRE
10. is visible as CRE to other staff members in the department
11. points out when educational projects succeed
12. constantly works to make education a common responsibility in the department
13. helps to use critical incidents as the basis for learning
14. is the first to use feedback as a natural part in daily work
15. makes sure that all doctors in the department teach
16. constantly spreads the use of supervision as a tool in specialist training
17. works towards making it attractive to work with education

### Management

### Administrative skills

The CRE is responsible for specialist training in the department. The function of the CRE concerns four areas: The department in general, the trainees, the supervisors and the clinical teachers.

*Tasks in relation to the department*

The CRE in your department

1. considers education in all procedural changes in the department
2. initiates new educational activities
3. makes sure that the educational programmes are in line with the educational offers in the department
4. involves relevant staff to meet the educational programmes
5. constantly coordinates the educational programmes and the working schedule
6. spreads new learning- and evaluation strategies in the department
7. follows up on results of total evaluations to optimize education in the department

*Tasks in relation to trainees*

The CRE in your department

1. makes sure that all educational programmes at the department are updated
2. keeps the department introduction programmes updated
3. ensures that the introduction programmes are implemented
4. is aware that the trainees have facilities for skills training
5. helps the trainee to access necessary functions in order to obtain competence according to their individual education plan
6. makes sure that daily clinical guidance and training takes place
7. makes sure that the assessment of trainee competencies takes place
8. focus on individual learning plans to secure quality
9. invites trainees to seek relevant training opportunities
10. actively intervenes in problematic educational courses
11. can answer questions about education

*Tasks in relation to the supervisors*

The CRE in your department

1. points out a supervisor for all trainees
2. precisely delegates tasks to the supervisors
3. ensure that supervisors have the necessary qualifications
4. makes enquiries on the results of all appraisal meetings
5. supervises the supervisors
6. makes sure the supervisors know the learning and assessment strategies described in the educational programmes and curricula
7. keeps the supervisors updated on their responsibilities and tasks
8. sees to it that supervisors develop as supervisors

*Tasks in relation to clinical teachers*

The CRE in your department

1. precisely delegates tasks to the clinical teachers
2. supervises the clinical teachers
3. ensure that the clinical teachers know the learning and assessment strategies described in the educational programmes and curricula
4. sees to it that the clinical teachers develop skills as teachers
5. keeps the clinical teachers updated on their responsibilities and tasks
